# Contact to Natives Among Recent Turkish Migrants in Germany: Gender Differences and Potential Explanations

**DOI:** 10.3389/fsoc.2020.00060

**Published:** 2020-08-21

**Authors:** Verena Seibel

**Affiliations:** Department of Sociology, Faculty of Behavioural and Social Sciences, University of Groningen, Groningen, Netherlands

**Keywords:** gender differences, migrants, contact to natives, family influence, opportunity structure, preferences

## Abstract

Migrant men and women still differ extensively in their integration chances within receiving societies. Research suggests that next to educational discrepancies and traditional gender roles, migrant men benefit particularly from their contact to natives who facilitate the access to other relevant resources such as employment. However, we know actually very little about how recent migrant men and women build their social networks within receiving societies, how their networks differ, and why they potentially differ. In this paper I therefore study Turkish migrants in Germany within their first years after migration and the extent to which Turkish men and women differ in their likelihood to have contact to natives. Theoretically, I explore three main determinants for potential gender differences: Family influence, opportunity structure, and personal preferences. I thereby make use of the two-wave data from the “Social Cultural Integration Processes” Project (SCIP) which studies migrants within their first 3 years after migration. I find that after 3 years after migration Turkish women are not only more likely to report to have no contact to natives than Turkish men; Even if they do have contact, this contact occurs significantly less frequent among Turkish women than among Turkish men. Results suggest that Turkish women, who migrated for family reasons are exposed to the influence of the family in the receiving country, which is often found to govern social behavior. Also, compared to Turkish men, Turkish women are less likely to be employed which limits their opportunity to meet natives. Gendered preferences for contact to natives, however, do not explain why Turkish women have less contact to natives than Turkish men.

## Introduction

Migrant men and women still differ extensively in their integration chances within receiving societies, particularly with regards to labor market integration (Khoudja and Fleischmann, 2015; Ala-Mantila and Fleischmann, 2018). Research suggests that next to educational discrepancies and traditional gender roles, migrant men benefit particularly from their contact to natives who facilitate the access to other relevant resources such as employment. Studies suggest that men and women indeed differ in their social behavior (Moore, 1990) and that also among migrants social ties are created differently by men and women (Hagan, 1998; Curran et al., 2006; Martinović, 2013). However, we know actually very little about how migrant men and women build their social networks within receiving societies, how their networks differ, and why they potentially differ.

In this contribution I study gender differences in contact to natives among Turkish migrants, who arrived only recently in Germany. Integration processes are path-dependent and inequalities at the beginning of migration cumulate over time (Fuller and Martin, 2012). Integration patterns within the first years of arrival require therefore special attention. Theoretically, I focus on Kalmijn's (1998) theoretical distinction of the following three main determinants of contact to natives: Family influence, opportunity structure, and preferences. Each of these dimensions are gendered and therefore likely to lead to different outcomes for migrant men and women with regards to contact to natives. First, migrant families govern social behavior of their family members, by supporting “good” ties and sanction perceived “bad” ties (Kalmijn, 1998). This process is gendered as migrant women are often exposed to gendered norms of social behavior within the family (Arends-Tóth and Van de Vijver, 2009; Röder and Mühlau, 2014), likely to promote co-ethnic contacts instead of contact to natives. Second, migrant women often have fewer opportunities to meet natives than migrant men due to limited access to relevant loci such as educational institutions or the work place (Kalmijn, 1998), but also due to lower language skills compared to migrant men (Haug, 2008). Third, literature suggests that migrant women might have weaker preferences for contact to natives than migrant men: Women generally express stronger preferences than men for close-knit social relations (Moore, 1990), which in the migrant context consist mainly of co-ethnic contacts rather than contacts to natives.

I test my assumptions using two-wave data from the German “Social Cultural Integration Processes” Project (SCIP) (Diehl et al., 2016), which was collected among others among Turkish migrants in Germany who migrated within the last 18 months upon the time point of the survey and who have been surveyed again after another 15 months. The data thereby captures a time period in migrants' migration experience which is crucial for their further integration chances into the receiving society (DiPrete and Eirich, 2006; DiMaggio and Garip, 2012; Fuller and Martin, 2012) and allows for cautious causality assumptions due to its panel structure.

I thereby compare three groups of migrants with each other: The first group indicates to spend almost never time with natives. The second group spends time with natives on a yearly or monthly basis and the third group even at a weekly or daily basis. My findings suggest that Turkish women and men differ quite extensively in their contact to natives. Around 3 years after migration, a significant share of Turkish women still has hardly any contact to natives. In addition, even if Turkish women do report to have contact to natives, they spend significantly less time with these natives than Turkish men. Results suggest that part of this gender difference can be explained by Turkish women being less likely to be employed than Turkish men which limits their opportunity to meet natives. Also, migrant women who migrate for family reasons are more exposed to the influence of the family in the receiving country. Migrant families are found to govern social behavior, particularly of their female family members (Parrado and Flippen, 2005). Gendered preferences for contact to natives, however, do not explain why Turkish women have less contact to natives than Turkish men. Last, but not least, this study shows that family migration is a strong barrier for female labor market participation, thereby hindering their social integration.

This contribution is structured as followed: After discussing the theoretical concept of contact with natives among migrants, I continue with describing the three main determinants by Kalmijn (1998) used to explain contact to natives: family influence, opportunity, and preferences. I then discuss how these factors are gendered and why we can expect different outcomes in contact to natives for migrant women and men, followed by a short discussion about how these factors are interrelated. This section is followed by a description of the data, measurements, and methods as well as the results of the analysis. The contribution finishes with a short summary and discussion of the main results and the societal implications of the findings.

## The Importance of Contact to Natives

With reference to Granovetter's concept of strong and weak ties (Granovetter, 1973), the migrant literature distinguishes between bridging and bonding ties. Bonding ties exist between members of the same ethnic group and are characterized by high level of group solidarity and trust (Portes and Sensenbrenner, 1993), whereas bridging ties to natives “cut across the ethnic divide and as that span structural holes” within networks (Lancee, 2012, p. 29). Contact to natives are therefore considered as “bridges” to the native society. Particularly in the field of labor market integration, bridging ties have gained prominence as they increase the chances of employment, a higher income, and a higher occupational status (e.g., Kanas et al., 2011; Lancee, 2012; Seibel and Van Tubergen, 2013; Griesshaber and Seibel, 2015). However, contacts to natives are not only perceived beneficial in terms of better job opportunities; they lead to a stronger identity with the receiving country society (Vroome et al., 2014) and generate interpersonal trust among different ethnic groups as well as reinforce community ties, by interconnecting people of different backgrounds (Putnam, 1993, 2000).

The existing literature on determinants of contact to natives has mainly focused on strong ties such as inter-marriage (Harris and Ono, 2005; Carol, 2014, 2016) and friendship with natives (Martinović et al., 2011; Schacht et al., 2014; Smith et al., 2014)^1^. This focus is certainly justified as strong ties to natives signal the most intimate relation and represent one of the final stages of assimilation (Gordon, 1964). However, the lack of friendships to natives among migrants does not necessarily indicate a lack of integration as migrants can still hold frequent and friendly relations to natives without being close friends. In this contribution I am therefore more interested in migrants' general level of contact with natives as the absence of such general contacts tells the even more important story: Migrants who report to have (almost) no contact to the native population do not just lack a native friend or a native spouse, they lack the most basic access to the receiving society, its people and culture. It is therefore crucial to study not only the emergence of strong ties to the native population, as done in previous research, but also to ask the simple question whether migrants have contacts with natives at all, and if so, at what intensity. I therefore focus on a looser definition of bridging ties, namely the frequency of time spend with natives, thereby following Lancee's (2012) conception of structural bridging ties of which the frequency of contact to the native population is a valid measurement.

In order to assess gendered dimensions of contact to natives I follow previous research and focus on Kalmijn's (1998) theoretical distinction between third party influence with a focus on the migrant family, opportunity structure, and preferences. I will first introduce these concepts, explaining how each of these factors impacts migrants' chances to engage in contact to natives. In a second step, I will further elaborate how these three dimensions are gendered and why we can therefore expect different outcomes in contact to natives for Turkish women than for Turkish men.

## Emergence of Contact to Natives: Family Influence, Opportunity Structure, and Preferences

The extent to which migrants get in contact with the native population depends, according to Kalmijn (1998), on three main factors: Third party influences, opportunity structure, and individual preferences. So-called third parties influence the extent to which migrants create and maintain ties to the native population by supporting “good” ties and sanctioning perceived “bad” ties (Kalmijn, 1998; Pettigrew, 1998). In this context, previous research has particularly emphasized the relevance of migrant families in exerting influence on social contact building by enforcing cultural-based norms of social behavior (Parrado and Flippen, 2005; Martinović et al., 2011; Schaeffer, 2013; Carol, 2014; Schacht et al., 2014). I will therefore speak in the following specifically about family influence. Particularly in collectivistic cultures, norms of endogamy are transmitted within migrant networks, encouraging particularly contact to co-ethnics rather than natives (Kalmijn, 1998).

Previous research has studied several aspects of *family influence* on migrants' social behavior. Martinović et al. (2011) find that migrants who migrate or reunite with their family are more exposed to norms encouraging co-ethnic relations and therefore have fewer opportunities for inter-ethnic contact to natives than migrants who arrived in the receiving country for work or education. One of the reasons is that family migration often leads to immediate legal dependency on the family members already residing in the receiving country with regards to resident and work permit. This in turn increases the family's negotiation power and influence with regards to family members' social relationship building (Boyd and Grieco, 2003). Research suggests that particularly Turkish families prefer co-ethnic contact over contact to natives. Carol (2014), for example, finds that Turkish parents exert strong influence on their children's friendship network composition with regards to ethnicity, favoring friendships to co-ethnics. Also, a co-ethnic partner decreases the likelihood of engaging in inter-ethnic friendships to natives compared to a native partner (Martinović et al., 2011). Hence, the likelihood of engaging in contact with the native population seems to be influenced by the presence of family within the host country.

Next to family influence, migrants need to have the opportunity to actually meet natives (Blau, 1977). Hence, depending on the *opportunity structure* within migrants' environment, migrants are more or less likely to get in contact with natives. Certain settings such as educational institutions and the workplace have thereby been identified as important loci for migrants to get in contact with the majority population (Kalmijn, 1998; Kalmijn and Flap, 2001; Mouw and Entwisle, 2006; Schroedter and Kalter, 2008). Hence, whereas previous research has mainly emphasized the importance of inter-ethnic contact for migrant labor market integration (for example, Kanas et al., 2011), the causal relation is likely to also go the other direction: Migrants who manage to find labor also increase their opportunities to get in contact to natives. Moreover, attending education in the host country has been found to lead to increasing contact with the native population (Kanas and Van Tubergen, 2009). Next to ethnic loci, language skills impact migrants' opportunity of getting in contact with natives. Host country language skills not only enable basic communication, they also decrease the social distance between ethnic groups (Bogardus, 1959; Portes and Rumbaut, 1996) which is an important predictor of inter-ethnic contact (Kashima and Loh, 2006).

Several studies have emphasized the significance of language for inter-ethnic contact. Martinović et al. (2009) find for the Netherlands, that migrants who speak the receiving country's language well develop more contact with the native population over time than migrants who lack these language skills. Lancee and Seibel (2014) also show for six European countries that language proficiency positively affects Turkish migrants' chances to receive visits from natives and Schacht et al. (2014) show for Germany that language skills increase the chance for inter-ethnic friendships between migrants of various backgrounds and natives.

Last but not least, individuals must have certain *preferences* for creating contact to a specific group. Most individuals prefer social relations with similar others (McPherson et al., 2001) with regards to the educational background, attitudes, but also ethnic background (Kalmijn, 1998). Research therefore suggests that migrants are likely to prefer co-ethnic contact over contact to natives, since the shared cultural background is also associated with shared values, resources, and tastes (Smith et al., 2014).

## Gender Differences

Since integration is a path-dependent process, already small gender inequalities within the first years of migration are likely to lead to larger gender gaps later in life. It is therefore important to understand how and why migrant men and women differ in their contact with natives within the first years after migration. Each of the dimensions mentioned above, family influence, opportunity structure, and preferences, are gendered and likely to lead to different outcomes for migrant women than for migrant men. With regards to family influence, research suggests that migrant women's likelihood of engaging in contact to natives is more strongly influenced by their families compared to male migrants. The mechanism is two-fold: First, norms of social behavior transmitted within migrant families are often gendered and promote co-ethnic contacts, while discouraging contact to natives, more strongly for women than for men (Arends-Tóth and Van de Vijver, 2009; Diehl et al., 2009; Röder and Mühlau, 2014). One of the reasons seems that Turkish women are often perceived a cultural transmitters by their families (Kalmijn and Van Tubergen, 2010), a notion that is found to be manifested and enforced by the migration process itself, which is often experienced as a disruptive intervention and therefore strengthens the desire for maintaining cultural traditions. As a consequence, “women's roles become the “bastion” of continuity and tradition by idealizing gender behavior” (Parrado and Flippen, 2005, p. 611). This is particularly true if women have frequent contact with their migrant family within the host country, as Parrado and Flippen (2005) show, since migrant families “add to women's domestic responsibilities, tend to reinforce more traditional family values, or are disproportionately skewed toward the husband's side of the family” (p. 628). Such a focus on maintaining the home country's culture might explain why, for example, Turkish women and daughters are more strictly monitored than men and sons (Idema and Phalet, 2007) in terms of, for example, partner choice (Carol, 2014), favoring co-ethnic partners over native partners.

Second, migrant women are more likely to select into migrant families within the receiving country than migrant men. This has, among others, to do with gendered differences in migration motives: Among migrant women in Germany, the dominant migration motive is family reunification, whereas a significant share of migrant men migrate alone in order to seek employment BAMF (2014). Although in the Turkish community the family is also an important pull factor for male migrants, women are still more affected. Turkish women therefore often immediately fall into the family's arms and are as a consequence often being “classified by their relation to men…” (Boyd and Grieco, 2003, p. 5). Because Turkish women are more likely to migrate to the receiving country for family reasons than Turkish men, they are more likely to be exposed to the social influence of their migrant families who, as discussed above, generally favor co-ethnic contact for their female family members over contact to natives. As a consequence, one can expect that Turkish women might be less likely to engage in contact to natives than Turkish men.

Gender differences are also found with regards to migrants' opportunity to meet and establish contact to natives. First, employment rates are significantly lower among migrant women than migrant men, particularly for third-country nationals such as Turkish migrants (Kogan, 2006). As a consequence, migrant women often miss one of the most important loci for meeting natives, namely the workplace (Hagan, 1998). This gender gap in employment opportunities can be explained by two main factors: First, female migrants remain responsible for care-taking activities at home such as child rearing (Parrado and Flippen, 2005). Secondly, and this relates to the first point: Migrant families often decide to invest first and foremost into men's human capital in the form of job-seeking or language acquisition since male family members are expected a higher pay-off on the labor market (Van Tubergen and Kalmijn, 2008), due to their higher skill level (Tansel, 2002; Gündüz-Hosgör and Smits, 2006). This human capital investment gap might also explain why Turkish women in Germany possess lower language skills than male Turkish migrants (Haug, 2008). Hence, we can assume that Turkish women are less likely to engage in contact to natives than Turkish men because they lack the opportunities to meet and engage with natives.

Last but not least, migrant men and women might also differ in their personal preferences to engage in contact to natives. Studying gender differences in formal participation in associations, Inglehart and Norris (2003) show that women tend to spend more time with their family and immediate relatives (strong ties) than men, independently from other factors such as their opportunity structure. Women generally seem to prefer small networks characterized by high levels of trust (Burt, 1998). Translated to the context of migration one can assume that migrant women might prefer social interaction within kin-based and trusted co-ethnic networks (Portes and Sensenbrenner, 1993) whereas migrant men also seek contact outside of the family or co-ethnic community.

Another aspect supporting this assumption can be found in the argument that preferences for certain social relations are also shaped by the perceived value of these contacts. If certain contacts enable the achievement of set goals than these contacts can be preferred over others (Schroedter and Kalter, 2008, p. 361). Migrant men and women might differ in what they perceive as valuable in a contact. Since migrant men are often interested in finding adequate employment in order to improve their family's living conditions, migrant men might have stronger preferences for contact to natives who are assumed to possess more information about the labor market, both in quantity and quality (Behtoui, 2008; Kanas et al., 2009; Lancee, 2012), and are better informed about job openings (Mouw, 2002). Turkish women are often not expected to enter the labor market and therefore might be less interested in inter-ethnic relations than men. Rather, Turkish women might prefer kin-based relations which are characterized by trust. Indeed, research in the Netherlands shows that Turkish women express stronger preferences for co-ethnic relations than Turkish men which leads to fewer interactions with the native population (Martinović, 2013).

Of course, we have to take into account that these three factors opportunity, family influence, and preferences are not independent and we can think of numerous possibilities how these factors influence each other. However, I would like to analyse their interdependence from the lens of path-dependency, arguing that particularly for migrant women, it matters whether they migrate to Germany for family reasons or not. The presence or absence of the migrant family at the beginning of migration is likely to impact migrant women's chances of employment but also formation of preferences. Families might influence the likelihood of migrant women participating on the labor market. Strong believes about traditional gender roles within a family, for example, might hinder newly arrived women to put effort into finding employment. Similarly, the need to learn the host country language might be less prevalent if the migrant family is present, particularly for women, who do not intend to enter the labor market. In addition, as argued above, migrant families might influence migrant women's investment in language skills since preference is given to male family members who are expected to provide for their family by entering the German labor market (Van Tubergen and Kalmijn, 2008). In addition, Migrants' preferences for contact with natives, e.g., are likely to be influenced by their family's norms of cultural interaction. This might be particularly true for migrant women who, as discussed above, are considered the “bastion” of culture (Parrado and Flippen, 2005). Migrant women might therefore adapt their preferences to the expectations their families have regarding their social behavior. I therefore expect family migration to influence migrants' chances of contact with natives, particularly for Turkish women, and that the effect of family migration is mediated by (Turkish women's) chances of having the opportunity to meet natives and preferences for contact with natives.

## Data, Measurements, and Methods

### Data

The analyses of this study are based on the two-wave data derived from the “Social Cultural Integration Processes” Project (SCIP) (Diehl et al., 2016). The data was collected via Computer Assisted Personal Interviews (CAPI) within both waves, combined with Computer Assisted Telephone Interviews (CATI) in the second wave. The Survey was conducted in the years 2010 and 2011, inter alia, among migrants from Turkey who migrated within the last 18 months to Germany, with a follow-up survey around 1.5 years later. For most migrants, little physical contact to Germany existed before migration. Over 80% of the respondents indicated that they have never visited Germany longer than 4 weeks before migrating to Germany.

All interviews were conducted in Turkish. The sample was randomly drawn from the population registers of five large cities (Berlin, Hamburg, Munich, Cologne, and Bremen). In total, 580 Turkish migrants between the age of 18 and 60 were interviewed in both waves (please see Gresser and Schacht 2015 for detailed description of the methodological setup of the project). After deleting missing cases on either the dependent or independent variables, 384 cases were left for the analyses.

This data is therefore one of the few that look at the socio-cultural integration of migrants, who only recently migrated to the host society. If we know what factors drive inequalities in the social integration of migrant men and women within the first years after migration, appropriate measures could possibly still be effective. Moreover, the data consists of two waves; all independent variables are taken from the first wave whereas the dependent variable is taken from the second wave. Although we cannot make clear statements regarding the causality of dependent and independent variables, the data certainly provides a better insight into the causal link between integration concepts than cross-sectional data.

### Measurements

Respondents were asked in both waves “How often do you spend time with people from Germany?” with answer categories ranging from 1 to 6 (1 “Never,” 2 “less often,” 3 “several times a year,” 4 “few times a month,” 5 “several times a week,” 6 “every day”). I regrouped the six categories into the following three: 1 (“never” and “less often”) 2 (“several times a year” and “a few times a month”) and 3 (“several times a week” and “every day”). Respondents who fall into the first category are particularly interesting since they indicate to have almost no contact to the native population. The outcome variable was taken from the second wave, whereas all independent variables are taken from the first wave.

*Family influence* is measured as follows: First, respondents were asked about their migration motive: “There are different reasons for moving to Germany. Why did you move?” Respondents could choose multiple answers: For work, education, marriage, joined other family member, moved together with family members, political reasons, and other reasons. This variable unfortunately does not reflect migrants' legal status, but the motive only. Since I am interested in the impact of the family, I regroup all migrants who mentioned, among others, marriage or family members as their migration motive, since this indicates the presence of the family in the host country (1). Migrants who did not mention family but work, education, political reasons or other reasons were regrouped to one category (0). In addition, I look at whether respondents report to have a partner with migration background. Respondents were asked whether they live with a partner and whether this partner was born in Germany or outside of Germany. I created a dichotomous variable with the outcomes migrant partner (1) and native partner/no partner (0). Of course, substantial differences might be present between migrants who have a native partner or no partner at all. However, particularly among migrant women only very few do not have a partner. Moreover, this coding allows me to study my main interest, namely whether migrants experience an influence from their migrant family. We still have to consider, though, that this measurement does not reveal whether partners born in Germany have a migration background themselves. I still refer to this group as “natives,” as they have been socialized in Germany and are likely to differ in many dimensions from people who have not been born in Germany.

*Opportunity* measures include employment status, language skills, and education received in Germany. Respondents were asked about their current main activity (1 = Employed, 0 = unemployed, 3 = in education, 4 = sick, 5 = retired, 6 = at home, 7 = other). Due to the general low number of cases and because I am mainly interested in the relation of labor market participation and contact with natives I created a dichotomous variable with 1 (= working) and 0 (= Not working). Respondents' language skills were operationalized by taking the mean of speaking, writing, understanding, and reading the host country's language (0 = very bad to 1 = very good). Respondents were also asked whether they have received education in Germany (0 = No, 1 = Yes, primary education, 2 = yes, lower/higher secondary education, 3 = yes, tertiary education). Since only a limited number of Turkish migrants attended education in Germany at all, I regrouped the variable into 0 (No education in Germany) and 1 (yes, received education in Germany). Moreover, respondents who indicated as main activity “in education” were also coded 1 for receiving education in Germany.

Respondents are also asked about their *preferences* regarding their social life by answering to the statement “I prefer social activities which involve 1 = both, people from receiving country (RC) and country of origin (CO); 2 = RC people only; 3 = CO people only; 4 = neither.” Since I am mainly interested in whether migrant men and women differ in their preferences for contact with natives, I regrouped these four categories into two categories with the outcomes 1 (prefer social activities that involve both ‘people from RC and CO’ or ‘RC people only’) and 0 (preference for social activities involving ‘CO people only’ or ‘neither’).

I further control for whether respondents have stayed in Germany for longer than 4 weeks before migrating to Germany (0 = No; 1 = Yes), their length of stay (in months), whether they have children (0 = no children; 1 = children), age, religiosity (1 = very religious to 4 = not religious at all), and for the respondents' highest education within the country of origin (1 = no/primary education, 2 = secondary education, 3 = tertiary education). Last, but not least, do all models contain the variable ‘contact with natives’ from the first wave [again with the three categories: 1 (“never” and “less often”) 2 (“several times a year” and “a few times a month”) and 3 (“several times a week” and “every day”)] in order to adjust for any bias resulting from social interaction within the first months after arrival.

### Method

The outcome variable consist of three categories and results are based on multinomial logistic regression analyses and presented as relative risk ratios (rrr). In principle, the categories are ordered which would call for ordered logistic regression. However, I am particularly interested in the group of migrants who indicate that they never or very rarely spend time with natives and how this group relates to migrants who report more frequent interaction with natives. Multinominal logistic regression allows for such comparisons and form the first step of the analyses. In a second step, I examine the extent to which the effect of gender is mediated by the trias family influence, opportunity, and preferences. This is the case if gender has a significant effect on the mediator in question and if the indirect effect of gender via the mediator is significant. I therefore estimate the effect of gender on each mediator variable (see **Table 5**) and, in a third step, conduct a decomposition analysis using the Karlson-Holm-Breen (KHB) method (see **Table 6**), which is developed for binary and logit probit models, but can also be applied to other non-linear probability models such as multinominal regression. The KHB thereby provides an unbiased decomposition of the total effect into a direct and an indirect effect (Kohler and Karlson, 2010).

Last, but not least, I study the interplay between family influence, opportunity, and preferences separately for Turkish men and women. I am particularly interested in whether migrant families influence migrant women and men's opportunity to meet natives as well as their preferences. Again, the analyses follow these three steps: First, I examine the direct effects of these variables on contact with natives using multinominal logistic regression analyses. Then I estimate the effect of the migrant family on opportunity and preferences followed by the KHB decomposition analysis.

Results are presented in the following order: Discriptives are to be found in Table 1 and main results in Tables 2–4. Estimations of the effect of the main independent variable on potential mediators are presented in Tables 5, 6 presents the results of the KHB decomposition analyses.

**Table 1:** Descriptives of main independent variable and control variables, by time spend with natives and gender.

	**(Almost) never**				**Yearly/monthly**						**Weekly/daily**					
	**Women**		**Men**		**Women**		**Men**				**Women**		**Men**				
	**Mean**	**SD**	**Mean**	**SD**	**Mean**	**SD**	**Mean**	**SD**			**Mean**	**SD**	**Mean**		**SD**	**Min**	**Max**
Reason for migration: Family	0.97		0.89			0.85		0.63			0.72		0.72		0	1	
Migrant partner	0.59		0.53			0.56		0.44			0.51		0.28		0	1	
Employed	0.07		0.37			0.09		0.22			0.20		0.41		0	1	
Language skills	2.08	0.55	2.28		0.56	2.35	0.66	2.32		0.63	2.42	0.59	2.37	0.58	1	4	
Education in RC	0.13		0.18			0.29		0.15			0.37		0.36		0	1	
Preferences for contact with natives	0.80		0.76			0.94		0.89			0.85		0.91		0	1	
Contact with natives _t−1_															
(Almost) never	0.54		0.47			0.35		0.33			0.25		0.10		0	1	
Yearly/monthly	0.11		0.18			0.24		0.22			0.11		0.07		0	1	
Weekly/daily	0.34		0.34			0.41		0.44			0.64		0.83		0	1	
Length of stay (months)	28.93	4.99	28.13		5.23	25.76	5.61	27.56		6.08	26.35	5.28	26.97	5.32	18	40	
Previous stay in RC	0.11		0.26			0.09		0.26			0.25		0.24		0	1	
Highest level of education in CO															
No/primary education	0.38		0.29			0.15		0.11			0.23		0.09		0	1	
Secondary education	0.34		0.39			0.47		0.41			0.32		0.38		0	1	
Tertiary education	0.28		0.32			0.38		0.48			0.45		0.52		0	1	
Children (=yes)	0.34		0.37			0.18		0.41			0.23		0.26		0	1	
Religiousity	2.13	0.88	2.68		0.93	2.41	1.05	2.41		1.08	2.52	1.06	2.61	0.90	1	4	
Age	30.41	7.70	32.03		7.94	29.09	6.67	32.22		8.02	29.95	7.28	29.57	6.74	19	60	
*N*	61		38			34		27			75		151				

**Table 2:** Multinominal Logistic Regression Analysis (Relative Risk Ratio): Impact of gender, family influence, opportunity, and preferences on time spend with natives.

	**(Almost) never vs. weekly/daily**					**Yearly/monthly vs. weekly/daily**				
	**A1**	**B1**	**C1**	**D1**	**E1**	**A2**	**B2**	**C2**	**D2**	**E2**
Gender: Female	2.37^**^	2.33^**^	2.06^*^	2.38^**^	2.11^*^	2.21^*^	2.19^*^	1.81+	2.18^*^	1.72+
	(3.10)	(2.85)	(2.50)	(3.11)	(2.38)	(2.55)	(2.51)	(1.79)	(2.49)	(1.65)
Reason for migration: Family		3.12^*^			2.24		0.72			0.36^*^
		(2.28)			(1.58)		(−0.82)			(−2.22)
Migrant Partner		1.01			0.99		1.28			1.47
		(0.04)			(−0.02)		(0.71)			(1.06)
Employed			0.53+		0.61			0.36^*^		0.25^**^
			(−1.81)		(−1.38)			(−2.52)		(−3.15)
Language Skills			0.81		0.78			1.32		1.34
			(−0.83)		(−0.95)			(0.98)		(1.05)
Education in RC			0.38^*^		0.45^*^			0.48+		0.34^*^
			(−2.56)		(−2.06)			(−1.93)		(−2.42)
Preferences for contact with natives				1.07	1.17				2.86+	2.93+
				(0.15)	(0.33)				(1.82)	(1.77)
Contact with natives_*t*−1_: (Almost) never (=ref.)	Ref.	Ref.	Ref.	Ref.	Ref.	Ref.	Ref.	Ref.	Ref.	Ref.
Yearly/monthly	0.58	0.55	0.64	0.57	0.59	1.31	1.35	1.29	1.06	1.09
	(−1.19)	(−1.27)	(−0.92)	(−1.21)	(−1.05)	(0.57)	(0.64)	(0.53)	(0.12)	(0.17)
Weekly/daily	0.18^***^	0.18^***^	0.20^***^	0.17^***^	0.20^***^	0.29^**^	0.30^**^	0.28^***^	0.22^***^	0.22^***^
	(−5.53)	(−5.42)	(−4.71)	(−5.11)	(−4.41)	(−3.21)	(−3.04)	(−3.31)	(−3.84)	(−3.68)
Length of stay in months	1.06^*^	1.05^*^	1.05^*^	1.05^*^	1.05^*^	0.98	0.99	0.98	0.99	0.98
	(2.28)	(2.09)	(2.10)	(2.24)	(2.03)	(−0.51)	(−0.46)	(−0.72)	(−0.42)	(−0.57)
Previous stay in RC	0.87	1.06	1.01	0.88	1.13	0.65	0.61	0.67	0.64	0.58
	(−0.39)	(0.15)	(0.03)	(−0.36)	(0.31)	(−1.14)	(−1.21)	(−0.95)	(−1.14)	(−1.21)
Highest education in CO: none/primary (=ref.)	Ref.	Ref.	Ref.	Ref.	Ref.	Ref.	Ref.	Ref.	Ref.	Ref.
Secondary education	0.58	0.60	0.57	0.58	0.59	1.73	1.69	1.75	1.88	1.85
	(−1.38)	(−1.29)	(−1.44)	(−1.39)	(−1.34)	(1.03)	(0.98)	(1.05)	(1.15)	(1.09)
Tertiary education	0.40^*^	0.52	0.43^*^	0.40^*^	0.51	1.31	1.21	1.45	1.38	1.27
	(−2.26)	(−1.60)	(−2.15)	(−2.27)	(−1.64)	(0.52)	(0.36)	(0.72)	(0.61)	(0.43)
Children (=yes)	0.99	0.95	0.78	0.99	0.80	0.95	0.92	0.83	0.95	0.76
	(−0.05)	(−0.16)	(−0.71)	(−0.03)	(−0.65)	(−0.13)	(−0.23)	(−0.47)	(−0.14)	(−0.68)
Religiosity	0.99	1.03	1.04	0.99	1.04	0.93	0.93	0.92	0.91	0.87
	(−0.07)	(0.18)	(0.27)	(−0.08)	(0.29)	(−0.47)	(−0.47)	(−0.51)	(−0.55)	(−0.78)
Age	1.02	1.01	1.01	1.02	1.01	1.02	1.02	1.03	1.02	1.02
	(0.97)	(0.57)	(0.59)	(0.93)	(0.46)	(1.11)	(0.83)	(1.12)	(1.05)	(0.96)
N	386	386	386	386	386	386	386	386	386	386
Pseudo–R2	0.1380	0.1492	0.1607	0.1438	0.1798	0.1380	0.1492	0.1607	0.1438	0.1798

*Relative Risk Ratio; t statistics in parentheses*.

+ *p < 0.10*,

* *p < 0.05*,

** *p < 0.01*,

*** *p < 0.001*.

**Table 3:** Multinominal Logistic Regression Analysis (Relative Risk Ratio): Impact of family influence, opportunity and preferences on time spend with natives, female sample.

	**(Almost) never vs. weekly/daily**					**Yearly/monthly vs. weekly/daily**				
	**F1**	**G1**	**H1**	**I1**	**J1**	**F2**	**G2**	**H2**	**I2**	**J2**
Reason for migration: Family	7.41^*^	9.62^**^	4.77+	7.29^*^	6.67^*^	1.62	1.55	1.28	1.53	0.93
	(2.33)	(2.62)	(1.82)	(2.30)	(2.11)	(0.70)	(0.62)	(0.32)	(0.59)	(−0.09)
Migrant Partner		0.42+			0.38+		1.11			1.56
		(−1.70)			(−1.93)		(0.22)			(0.90)
Employed			0.39		0.41			0.52		0.40
			(−1.44)		(−1.29)			(−0.77)		(−1.05)
Language Skills			0.53		0.45+			1.18		1.18
			(−1.62)		(−1.88)			(0.39)		(0.37)
Education in RC			0.52		0.46			0.83		0.71
			(−1.21)		(−1.48)			(−0.33)		(−0.57)
Preferences for contact with natives				1.38	1.43				5.34+	6.05+
				(0.52)	(0.55)				(1.89)	(1.87)
Contact with natives_*t*−1_: (Almost) never (=ref.)	Ref.	Ref.	Ref.	Ref.	Ref.	Ref.	Ref.	Ref.	Ref.	Ref.
Yearly/monthly	0.55	0.44	0.63	0.52	0.48	2.23	2.42	2.10	1.82	1.99
	(−0.88)	(−1.22)	(−0.67)	(−0.95)	(−1.10)	(1.18)	(1.23)	(1.10)	(0.89)	(1.03)
Weekly/daily	0.37^*^	0.30^**^	0.55	0.34^*^	0.42	0.48	0.52	0.47	0.34+	0.39
	(−2.38)	(−2.74)	(−1.25)	(−2.36)	(−1.64)	(−1.35)	(−1.19)	(−1.39)	(−1.92)	(−1.64)
Length of stay in months	1.08^*^	1.08^*^	1.08^*^	1.08^*^	1.09^*^	0.97	0.97	0.96	0.97	0.96
	(2.04)	(2.09)	(2.04)	(2.03)	(2.07)	(−0.77)	(−0.74)	(−0.83)	(−0.63)	(−0.72)
Previous stay in RC	0.72	0.73	0.91	0.70	0.96	0.33+	0.32+	0.33+	0.29^*^	0.25^*^
	(−0.59)	(−0.53)	(−0.16)	(−0.65)	(−0.07)	(−1.75)	(−1.89)	(−1.69)	(−1.98)	(−2.18)
Highest education in CO: none/primary (=ref.)	Ref.	Ref.	Ref.	Ref.	Ref.	Ref.	Ref.	Ref.	Ref.	Ref.
Secondary education	0.72	0.72	0.68	0.72	0.63	2.00	1.94	2.04	2.14	2.01
	(−0.67)	(−0.66)	(−0.74)	(−0.67)	(−0.86)	(1.06)	(1.03)	(1.07)	(1.11)	(1.04)
Tertiary education	1.06	0.92	0.98	1.06	0.82	1.65	1.59	1.76	1.81	1.96
	(0.12)	(−0.15)	(−0.05)	(0.11)	(−0.36)	(0.72)	(0.67)	(0.80)	(0.81)	(0.90)
Children (=yes)	1.05	1.43	0.89	1.05	1.21	0.68	0.64	0.71	0.64	0.55
	(0.11)	(0.71)	(−0.23)	(0.11)	(0.35)	(−0.64)	(−0.73)	(−0.52)	(−0.73)	(−0.89)
Religiosity	0.89	0.91	0.89	0.89	0.93	1.03	1.03	0.97	1.04	0.97
	(−0.57)	(−0.44)	(−0.55)	(−0.54)	(−0.31)	(0.10)	(0.10)	(−0.10)	(0.15)	(−0.12)
Age	0.99	1.00	0.99	0.99	1.00	0.99	0.99	1.00	0.99	0.99
	(−0.29)	(0.11)	(−0.30)	(−0.33)	(−0.07)	(−0.27)	(−0.30)	(−0.11)	(−0.35)	(−0.38)
N	170	170	170	170	170	170	170	170	170	170
R2	0.1254	0.1366	0.1463	0.1388	0.1768	0.1254	0.1366	0.1463	0.1388	0.1768

*Exponentiated coefficients; t statistics in parentheses*.

+ *p < 0.10*,

* *p < 0.05*,

** *p < 0.01*, ^***^p < 0.001.

**Table 4:** Multinominal Logistic Regression Analysis (Relative Risk Ratio): Impact of family influence, opportunity and preferences on time spend with natives, male sample.

	**(Almost) never vs. weekly/daily**					**Yearly/monthly vs. weekly/daily**				
	**K1**	**L1**	**M1**	**N1**	**O1**	**K2**	**L2**	**M2**	**N2**	**O2**
Reason for migration: Family	1.94	2.06	1.73	2.04	1.71	0.36^*^	0.36+	0.06^***^	0.37+	0.06^***^
	(1.10)	(1.23)	(0.90)	(1.21)	(0.88)	(−1.96)	(−1.91)	(−3.72)	(−1.87)	(−3.66)
Migrant Partner		2.85^*^	2.85+	2.84^*^	2.85+		1.51	1.54	1.50	1.51
		(1.99)	(1.95)	(1.99)	(1.95)		(0.72)	(0.77)	(0.73)	(0.72)
Employed			0.69		0.69			0.08^***^		0.08^***^
			(−0.68)		(−0.69)			(−3.65)		(−3.63)
Language Skills			0.96		0.98			1.73		1.65
			(−0.09)		(−0.06)			(1.28)		(1.17)
Education in RC			0.50		0.50			0.07^***^		0.07^***^
			(−1.06)		(−1.06)			(−3.41)		(−3.41)
Preferences for contact with natives				0.90	0.97				2.21	1.81
				(−0.16)	(−0.05)				(0.99)	(0.72)
Contact with natives_*t*−1_: (Almost) never (=ref.)	Ref.	Ref.	Ref.	Ref.	Ref.	Ref.	Ref.	Ref.	Ref.	Ref.
Yearly/monthly	0.44	0.49	0.53	0.51	0.54	0.72	0.74	0.66	0.59	0.57
	(−1.21)	(−1.04)	(−0.89)	(−0.94)	(−0.83)	(−0.50)	(−0.46)	(−0.57)	(−0.77)	(−0.73)
Weekly/daily	0.08^***^	0.07^***^	0.07^***^	0.08^***^	0.07^***^	0.14^***^	0.14^***^	0.10^***^	0.11^***^	0.08^***^
	(−5.08)	(−4.75)	(−4.49)	(−4.11)	(−3.99)	(−3.48)	(−3.50)	(−3.99)	(−3.81)	(−3.93)
Length of stay in months	1.02	1.01	1.01	1.01	1.01	1.03	1.02	1.03	1.03	1.03
	(0.65)	(0.27)	(0.19)	(0.24)	(0.17)	(0.61)	(0.50)	(0.63)	(0.54)	(0.66)
Previous stay in RC	1.60	1.95	2.13	1.94	2.12	1.01	1.06	0.96	1.11	0.99
	(0.79)	(1.07)	(1.22)	(1.06)	(1.19)	(0.01)	(0.09)	(−0.06)	(0.17)	(−0.01)
Highest education in CO: none/primary (=Ref.)	Ref.	Ref.	Ref.	Ref.	Ref.	Ref.	Ref.	Ref.	Ref.	Ref.
Secondary education	0.31+	0.25+	0.25^*^	0.25+	0.24^*^	0.89	0.82	0.98	0.92	1.02
	(−1.86)	(−1.90)	(−2.01)	(−1.95)	(−2.05)	(−0.14)	(−0.24)	(−0.02)	(−0.10)	(0.02)
Tertiary education	0.18^*^	0.16^*^	0.17^*^	0.16^*^	0.16^*^	0.64	0.60	0.46	0.63	0.47
	(−2.52)	(−2.40)	(−2.33)	(−2.41)	(−2.35)	(−0.57)	(−0.64)	(−0.87)	(−0.57)	(−0.83)
Children (=yes)	0.74	0.73	0.63	0.73	0.64	1.59	1.60	1.38	1.63	1.35
	(−0.63)	(−0.62)	(−0.84)	(−0.63)	(−0.83)	(0.88)	(0.89)	(0.56)	(0.92)	(0.53)
Religiosity	1.09	1.00	1.03	1.01	1.03	0.77	0.77	0.74	0.74	0.72
	(0.42)	(0.02)	(0.12)	(0.07)	(0.15)	(−1.11)	(−1.12)	(−1.08)	(−1.26)	(−1.16)
Age	1.03	0.99	0.99	0.99	0.99	1.04	1.02	1.05	1.02	1.05
	(0.85)	(−0.28)	(−0.35)	(−0.28)	(−0.36)	(1.51)	(0.72)	(1.21)	(0.63)	(1.12)
N	216	216	216	216	216	216	216	216	216	216
R2	0.1843	0.1971	0.2593	0.2009	0.2611	0.1843	0.1971	0.2593	0.2009	0.2611

*Relative Risk Ratio; t statistics in parentheses*.

+ *p < 0.10*,

* p < 0.05, ^**^p < 0.01,

*** *p < 0.001*.

**Table 5:** Impact of gender (whole sample) and family migration (women and men separately) on mediating variables.

**Mediating variable**	**Impact of gender, whole sample**	**Impact of family migration, female sample**	**Impact of family migration, male sample**
Family migration	1.49	–	–
	(0.47)	–	-
Migrant partner	2.50^***^	4.04	0.91
	(0.64)	(3.63)	(0.39)
Employment	0.25^***^	0.14^**^	0.63
	(0.07)	(0.09)	(0.24)
Language skills	0.03	−0.14	0.09
	(0.06)	(0.17)	(0.09)
Education in RC	0.94	0.09^***^	0.29^**^
	(0.24)	(0.06)	(0.11)
Preferences	1.31	2.13	0.69
	(0.48)	(1.67)	(0.47)

*Controlled for contact to natives_t−1_, length of stay, stay in RC before migration, education, children, age*;

*Standard errors in parantheses*;

+p < 0.10, ^*^p < 0.05,

** *p < 0.01*,

*** *p < 0.001*.

*Logistic regression for migrant partner, employment, education in RC and preferences for natives (Odds Ratio); Linear regression for language skills (beta)*.

**Table 6:** KHB Decomposition analysis: Significance (*p*-value) of indirect effect of gender (whole sample) and family influence (women and men separately) via mediating variables.

	**Impact of gender, whole sample**		**Impact of family migration, female sample**		**Impact of family migration, male sample**	
	**(Almost) never vs. weekly/daily**	**Yearly/monthly vs. weekly/daily**	**(Almost) never vs. weekly/daily**	**Yearly/monthly vs. weekly/daily**	**(Almost) never vs. weekly/daily**	**Yearly/monthly vs. weekly/daily**
Family migration and migrant partner	0.592	0.605	-	-	-	-
Migrant partner	-	-	0.184	0.824	0.676	0.713
Employment, language skills, education in RC	0.141	0.024^*^	0.06^*^	0.516	0.257	0.002^**^
Preferences	0.886	0.576	0.658	0.448	0.888	0.668

*Controlled for contact to natives_t−1_, length of stay, stay in RC before migration, education, children, age; ^+^p < 0.10*,

* *p < 0.05*,

** p < 0.01, ^***^p < 0.001.

## Results

**Figure 1:**
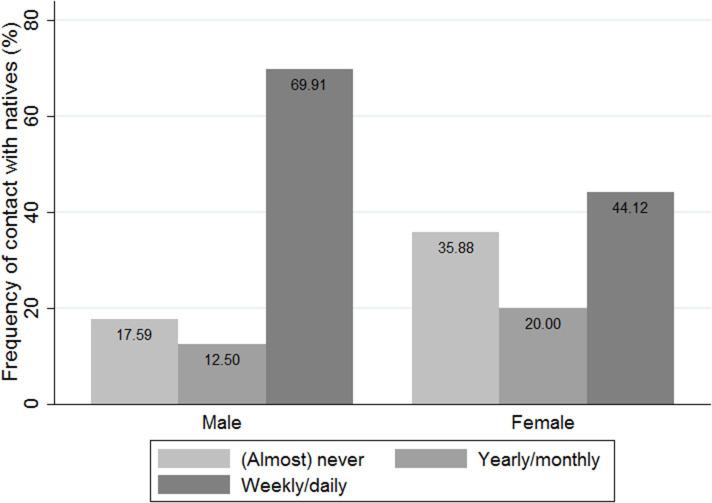
Frequency of contact with natives (%), by gender.

Figure 1 presents the percentages of Turkish migrant women and men who report to have no to hardly any contact to natives. Three years after migration, over 36% of Turkish women report that they spend no to hardly any time with natives, compared to 18% of Turkish men. Gender differences can also be found among those, who report to have at least some contact to natives: Almost 70% of Turkish men report that they spend time with natives on a weekly or daily basis, which is only the case for 44% of Turkish women. These descriptives therefore suggest that Turkish women are not only less likely to have contact to natives in the first place; once contact is established, Turkish women report lower frequencies of contact than Turkish men.

Table 1 presents further descriptives for Turkish women and men, distinguishing between those who report to spend hardly any time with natives (no contact), those migrants who report to spend time with migrants on a yearly or monthly basis and those who spend time with natives on a weekly or daily basis. The large majority of both men and women migrated to Germany for family reasons, though the numbers are higher for Turkish women. Also, among all three groups, the majority of Turkish women indicates to have a migrant partner, which is less the case for Turkish men.

Stronger differences between Turkish men and women are found with regards to employment. Among migrants who hardly spend any time with natives, only 7% of Turkish women are employed compared to 37% of Turkish men. Among those who spend time with natives on a weekly or daily basis, already 20% of Turkish women are employed, compared to 41% of Turkish men. Language skills are quite evenly distributed between men and women with the exception of migrants who hardly spend any time with natives. In this group, men report better language skills than women. Also, education in Germany has been followed by only 13% of Turkish women and 18% of Turkish men in the group who spends hardly any time with natives compared to 37 and 36%, respectively among those who spend time with natives on a weekly or daily basis. Regarding preferences, the majority of both, women and men, indicate to prefer spending time with German natives, though no gender-related pattern is observable across the three groups. Last but not least, in all three groups Turkish men are higher educated, slightly older and less religious than Turkish women.

The descriptive statistics already indicate gender differences in contact to natives. In the following I examine potential explanations for this gender gap. I conduct a multinominal logistic regression and compare migrants who indicated to (almost) never spend time with natives (group 1) and migrants who indicated to spend time with natives on a yearly/monthly basis (group 2) with those migrants who indicated that they spend time with natives on a weekly or daily basis (group 3). I start by examining the gender difference between migrants who have hardly any contact with natives (group 1) compared to migrants who interact with natives very frequently (group 3). Relative risk ratio's (rrr) are presented in Table 2 (model A1 to E1), the effect of gender on the mediator variables in Table 5 and the significance of decomposition analyses in Table 6. Migrant women have a 2.37 higher relative risk than men to almost never spend time with natives (model A1). Following Kalmijn et al.'s distinction between family influence, opportunity structure, and preferences, I continue examining this gender effect. First, one can assume that Turkish women are more likely to have no contact to natives due to their stronger embeddedness within their migrant family, which prefers co-ethnic contact over contact to natives for their female family members. In model B1, I therefore examine the potential mediating effect of partnership status and migrants' migration motive (whether family was the main motive), both factors expected to increase family influence on migrants' social behavior. However, the gender effect hardly changes between model A1 and B1. In Table 5 we see that gender indeed has no effect on the likelihood to migrate to Germany for family reasons, but that women are significantely more likely to have a migrant partner then men. However, the decomposition analysis (Table 6) shows that the indirect effect of gender on contact with natives via family migration and migrant partner is not significant (KHB *p* = 0.592). However, two points should be noted here. First, family migration and partnership status are highly correlated. Once, partnership is taken out of the model, family migration becomes significant (rrr = 3,10, *p* = 0.023; not presented in table); this is not surprising as most migrants who migrate for family reasons are married with the majority of migrant women being married to partners with a migration background, whereas Turkish men also being engaged with native partners. I will come back to this observation when estimating the effects of opportunity, preferences, and family influence separately for Turkish women and men (Tables 4, 5). Second, the effect of family migration does mediate the relationship between gender and the relative risk of spending almost no time with natives if the measurement contact with natives_t−1_ is taken out of the model. This suggests that family embeddedness has an effect especially in the first few years with later-ripening consequences. Women who do have little contact with natives because they migrated with or to their family remain to have little contact with natives 1 ½ years later. Because migration in most cases occurs before establishing contact with natives, we can assume a causal effect here.

I continue with adding migrants' employment status, language skills, and education in Germany to the model (model C1). I assumed that because Turkish women generally score lower on these factors than Turkish men, they have fewer opportunities to meet natives. We first look at the direct effects of these factors on migrants' relative risk of spending almost no contact to natives: Migrants who are employed (rrr = 0.53, *p* ≥ 0.10) and/or who followed (part of) their education in Germany (rrr = 0.381, *p* ≤ 0.01) are significantly less likely to have little contact to natives. Language skills also decrease the relative risk to spend little time with natives, though the effect is not significant (rrr = 0.808, *p* > 0.10). The next question is whether these human capital factors mediate the gender effect. The gender coefficient drops from 2.37 to 2.06 and Turkish women are indeed significantly less likely to be employed than men (Table 5), though no gender differences are found for language skills and education in Germany. Although gender influences the likelihood of employment, the indirect effect of gender via employment, language skills, and education in Germany is not significant in this model (Table 6, KHB *p* = 0.141). Hence, neither employment not language skills or education in Germany mediate the effect of gender on the likelihood of spending almost no time with natives compared to spending time with natives on a daily basis.

Last, but not least I study whether potential gender differences in preferences for contact to natives might explain why Turkish women report a higher risk of spending almost no time with natives (Model D1). The gender coefficient hardly changes and we also do not observe a significant effect of gender on preferences (Table 5). In addition, the indirect effect of gender via preferences is unsurprisingly not significant (Table 6, KHB *p* = 0.886). In the final model (Model E1), all explanatory variables are included. We can conclude that the gender effect remains strong and significant and is not mediated by the migrants' opportunity, preferences, and family influence. Further, we see that Turkish migrants with tertiary education have a lower relative risk to have no contact with natives. Children, religiosity, and age, however, have no effect on non-contact to natives.

I continue by comparing the relative risk of spending time with natives yearly or monthly compared to spending time with natives on a weekly or daily basis (Model A2 to E2). Again, I find Turkish women to be more likely to have contact with natives only a few times per year/months than Turkish men (rrr = 2.21, *p* ≤ 0.05). The gender effect hardly changes when including family influence measurements into the model (model B2, rrr = 2.19) and the indirect effect of gender via family influence is not significant (Table 6, KHB *p* = 0.605). However, when including employment, language skills and education in Germany (model C2), the gender coefficient drops from 2.21 to 1.81 and loses significance. We know that Turkish women are indeed less likely to be employed than Turkish men (see Table 5) and the decomposition analysis reveals that the indirect effect of gender on spending time with natives on a yearly/monthly basis compared to weekly/daily is significant (KHB *p* = 0.024). Hence, Turkish women indeed face a higher risk than Turkish men to spend time with natives only a few times per year or per month (compared to weekly or daily) because of their lower chances to be employed on the labor market. Last, but not least, I study whether preferences mediates the gender effect, however, neither does the gender effect change much, nor does the KHB decomposition analysis shows significance (KHB *p* = 0.576).

In a second step, I analyse all multinomial logistic regression models for Turkish men and women separately in order to understand the interplay between family influence, opportunity structure, and preferences (Tables 3, 4). I argue that we first have to look at the conditions under which migrants enter the receiving country. Migrants who migrate for family reasons are immediately embedded within their migrant family, which will have different impact on their social relationship building than if migrating to Germany without any family ties. I will first discuss the results for the female sample (Table 3, models F1 to J2) before continuing with the male sample (Table 4, models K1 to O2). We see that indeed for women family migration significantly increases the likelihood of spending almost no time with natives compared to spending time with natives on a weekly or even daily basis (model F1, rrr = 7.41, *p* ≤ 0.05). Model G1 then includes migrant women's partnership status and we observe two surprising results. First, migrant women who have a migrant partner have a *lower* risk to have almost no contact with natives than women who do not have a migrant partner whereas the opposite is the case for migrant men (Table 4, model L1). One explanation could be that migrant women use the ties of their migrant partners to get in contact with natives whereas migrant men do not have the same opportunities provided by their female partners. Still, this result remains puzzling and should be investigated further in future research. Second, the coefficient for family migration increases and not decreases. Further mediation analyses show that family migration does not significantly influence the likelihood of having a native partner for migrant women (Table 5) and that the indirect effect of family migration via having a migrant partner is not significant (KHB *p* = 0.184). In a second step I test whether family migration influences the likelihood of acquiring human capital in the receiving country, which in turn impacts migrant women's risk of having hardly to no contact with natives. Indeed, the family migration motive coefficient drops from 7.41 to 4.77 and becomes less significant. Migrant women who migrated for family reasons are less likely to be employed and less likely to have followed education in Germany than migrant women who did not migrate for family reasons (Table 5). Also, the KHB decomposition analysis shows that family migration has indeed a significant indirect effect on spending almost never time with natives (compared to spending time on a weekly or daily basis) (KHB *p* = 0.06). Hence, employment and education in Germany mediate the relationship between family migration and spending almost no time with natives. Preferences, on the other hand have neither any effect on the likelihood to spend little time with natives, nor does it mediate the relationship between family migration and contact with natives.

Models F2 to J2 examine the same pattern, this time comparing migrant women who indicate to spend time with natives yearly/monthly to women who spend time with natives on a weekly or daily basis. In these models we actually observe no effect of family migration. Also, employment, language skills, and education in Germany do not impact this relationship. Only preferences for natives increases the likelihood of spending time with natives only on a yearly/monthly basis compared to on a weekly/daily basis. This is surprising as we would expect the exact opposite, namely that migrant women who prefer spending time with natives have more contact than migrant women who do not express a strong preference for contact with natives. Again, we can only speculate, but one explanation could be that this result reflects the women's unrealized wish to spend more time with natives whereas women who do have frequent contact with natives are more neutral in this regard.

I now turn to the results for the male sample in Table 4. Neither family migration nor employment, language skills, education in Germany and preferences for natives increase the likelihood for migrant men to spend almost no time with natives compared to spending time with natives on a weekly or daily basis. Only among those who report to spend time with natives several times a year or month, we observe that family migration plays a role (model K2 to O2). Interestingly, Turkish men who migrated for family reasons are less likely to report to have contact with natives only occasionally instead of weekly/daily (model K2, rrr = 0.36, *p* ≤ 0.10). One explanation could be that migrant families purposely encourage contact to natives for their male family members in order to increase their labor market chances, whereas Turkish men with no family relations in the receiving country might lack these broker ties. In model M2 I include male migrants' employment status, their language skills and education in Germany. Employment status and education followed in Germany indeed decreases the relative risk of spending time with natives only on a monthly/yearly basis compared to a weekly/daily basis. Interestingly, the effect of family migration becomes stronger (rrr = 0.06, *p* ≤ 0.001). For Turkish men, family migration indeed lowers the chances of following education in Germany though not significant effect can be found for employment or language skills (Table 5). Also, the KHB composition analysis reveals that for men family migration has a significant indirect effect on the likelihood of spending time with natives on a yearly/monthly basis vs. on a weekly/daily basis via the opportunity factors. Given that family influence only affects Turkish men's likelihood of attending education in Germany, we can assume that this indirect effect can be mainly attributed to the variable education in RC. Adding preferences to the model (model N2 and O2) shows hardly any changes. Also, the effect of employment and education in Germany do not change when taking preferences into account which suggests that there is little correlation between these factors.

## Conclusion

Contact to natives among migrants in Europe has received increasing interest from the scientific community, mainly because of their beneficial impact on other integration dimensions such as the labor market. However, despite the valuable research in this area, little is known about gender differences in this regard. This is surprising as the outcomes of contact to natives vary tremendously between migrant men and women (e.g., Lancee, 2012) and therefore call out for a substantive research of the mechanisms of gendered relation-building.

This paper therefore contributes to the existing literature by looking at potential gender differences in contact to natives among recent Turkish migrants in Germany who have been staying in Germany for about 3 years at the time of the survey. Using unique two-wave data from the SCIP project I aimed at answering the question to what extent Turkish men and women differ in their contact to natives and why. I thereby compare migrants who indicate to spend almost never time with natives and migrants who report to spend time with natives on a yearly or monthly basis with natives who spend regularly time with natives, namely on a weekly or even daily basis. Results show significant differences between Turkish men and women. After 3 years of migration, 36% of Turkish women report to spend almost no time with natives, compared to 18% of Turkish men. These numbers are quite alarming as they show that Turkish women are not only less likely to engage in inter-ethnic partnership or friendship, as previous research has shown (Schacht et al., 2014; Carol, 2016); their complete lack of contact to natives indicates the absence of the most basic access to the receiving society, its people and culture.

How can we explain these gender differences? Accoding to Kalmijn (1998), contact to natives depends on three main factors: Family influence, opportunity structure, and preferences. All three dimensions are gendered and might explain why Turkish women have less contact to natives than Turkish men. First, migrants establish contacts under the influence of family members, which, through methods of social sanctioning and rewarding, govern their social behavior (Parrado and Flippen, 2005). However, Turkish women are more exposed to these family norms due to higher family migration and because Turkish women are more likely to engage in co-ethnic partnerships. Second, Turkish women often lack the opportunity to meet natives since they are less likely to participate in native-dominated loci such as the labor market and, related to this aspect, also less likely to learn the language of the receiving country sufficiently (Haug, 2008), which decreases their chances of engaging with natives. Third, contact to natives also depends on the personal preference, independent of people's opportunities and the influence of third parties. Whereas, previous research suggests that men and women might differ in their social preferences (Inglehart and Norris, 2003), little is known whether this is also true for migrant populations with regards to contact to natives. However, one can argue that Turkish men might have stronger preferences for contact to natives than Turkish women, since they benefit more from these relations with regards to their labor market integration.

Results of this study indicate that whereas personal preferences for contact to natives are neither strongly gendered, they also do not explain why Turkish women have less contact to natives than Turkish men. The family, however, seems to play a role, particularly for women. Turkish women who migrated to Germany for family reasons are more likely to spend almost no time with natives than Turkish women who migrate for economic or educational reasons. The data suggests that Turkish women who migrate for family reasons are less likely to enter the labor market than Turkish women who migrate for other reasons, which lowers their chances of meeting natives. However, although family migration impacts women's risk of having no contact with natives, it does not mediate the effect gender has on contact with natives. Similarly, I did not find confirmation for the assumption that advantageous opportunity structures in terms of employment, language skills, and followed education in Germany, nor gender-specific preferences for contact with natives explain why Turkish women have such a higher risk to spend almost no time with natives compared to Turkish men. It seems like this gender difference is set in stone, and future research has to pay more attention to this group of migrant women who seem to experience social isolation from the native society.

Comparing Turkish migrants who spend time with natives on a yearly or monthly basis to migrants who spend time with natives on a weekly or even daily basis, a strong gender difference is observable, too. However, part of this gender difference can be explained by Turkish women's lower chances to be employed on the labor market, which serves as an important loci to meet and interact with natives (Kalmijn, 1998). These results suggest that it is indeed worthwhile investing in female labor market participation, not only in order to increase their financial independence, but also to strengthen Turkish women's social integration into the society. However, this seems only to be the case for Turkish women who have already a certain amount of contact with natives. For women who report to have hardly no contact with natives, neither labor market participation nor improving language skills would increase the likelihood of having increased contact with natives. We have to consider that not all relevant factors were captured by the data. We do not know, for example, to what extent migrant families actually differ in their influence on their family members' social behavior. It could be, for example, that those Turkish women who report to spend almost no time with natives are embedded within specific family structures which make contact with natives less likely. Reasons can be driven by cultural differences, but also the size of the family might matter, since larger families might be more likely to fulfill the need for emotional and informational support than smaller families, thereby decreasing the need for inter-ethnic contact.

Of course, this study also contains other limitations, which need to be addressed. First, this study examines contact to natives by looking at the time migrants spent with natives. Such a frequency measure does not indicate whether migrants spend time with only a few or many natives and it is open to debate, whether the frequency matters for a successful social integration or the amount of people. Most likely, it is both. In addition, compared to other research studying contact to natives with a strong focus on inter-ethnic marriage or friendship, spending time with natives is a rather broad measure. However, it contains a valuable advantage as it is able to depict social marginalization. If migrants report to spend no time at all with natives but only with co-ethnics than this is alarming since it indicates the absence of any participation of societal life that includes natives in the receiving country. I find that Turkish women are twice as likely to report spending never time with natives than Turkish men. Hence, the absence of contact to natives is a women's issue which needs to be addressed by policy makers. Though this study does not provide explicit solutions for this specific group, we did learn that for Turkish women who already have some contact to the native population one of the most effective measures to further increase their social integration would be to increase migrant women's labor market participation. However, policy makers should also be aware of the extent to which migrant women are embedded within their families and the gender-specific norms they inhibit. Policy makers should therefore initiate gender-sensible programs, which increase inter-ethnic contact to natives among migrant women. This could be done, for example, by creating networks targeting cross-cultural exchange in form of women groups; particularly for migrant women who originate from countries, which are characterized by gender segregation in social life, women groups can establish a trusting network thereby contributing to inter-ethnic contact.

Second, one should consider that people might differ in their definition of the concept “spending time with someone.” Whereas, for some this implies a genuine exchange of time and information, others might think of the daily chat with the supermarket cashier. However, it is exactly this subjectivity of this measurement, which makes it so interesting. People simply perceive inter-actions and social exchange differently (Furman and Buhrmester, 1992) and its the perceptions which govern attitudes and behavior, not so much the objective fact.

Third, the data consists only of Turkish migrants living in one of the five largest cities in Germany and results are therefore not representative. Social behavior among migrants living in rural areas is likely to be different from the social behavior of urban migrants. However, we should note that the large majority of Turkish migrants indeed lives in urban, and not rural, areas.

Lastly, this study only looks at recent migrants in Germany. Although the first years after migration have been shown to be crucial for further integration (Fuller and Martin, 2012), one could argue that the initial gender gap in social integration between Turkish men and women might vanish over time. Turkish women might invest in their human capital after their male family members have been settled in the labor market thereby increasing their chances of inter-ethnic contact. Future research should therefore investigate gender differences in inter-ethnic relations over a longer period of time.

## Data Availability Statement

Data available at GESIS, see Diehl et al., 2016.

## Author Contributions

The author confirms being the sole contributor of this work and has approved it for publication.

## Conflict of Interest

The author declares that the research was conducted in the absence of any commercial or financial relationships that could be construed as a potential conflict of interest.

## Footnotes

^1^Strong ties stand in contrast to weak ties which are described as loose connections to a set of different individuals such as colleagues or acquaintances (Granovetter, 1973).
